# Universal Entropy of Word Ordering Across Linguistic Families

**DOI:** 10.1371/journal.pone.0019875

**Published:** 2011-05-13

**Authors:** Marcelo A. Montemurro, Damián H. Zanette

**Affiliations:** 1 The University of Manchester, Manchester, United Kingdom; 2 Consejo Nacional de Investigaciones Científicas y Técnicas, Centro Atómico Bariloche and Instituto Balseiro, San Carlos de Bariloche, Argentina; Queensland Institute of Medical Research, Australia

## Abstract

**Background:**

The language faculty is probably the most distinctive feature of our species, and endows us with a unique ability to exchange highly structured information. In written language, information is encoded by the concatenation of basic symbols under grammatical and semantic constraints. As is also the case in other natural information carriers, the resulting symbolic sequences show a delicate balance between order and disorder. That balance is determined by the interplay between the diversity of symbols and by their specific ordering in the sequences. Here we used entropy to quantify the contribution of different organizational levels to the overall statistical structure of language.

**Methodology/Principal Findings:**

We computed a relative entropy measure to quantify the degree of ordering in word sequences from languages belonging to several linguistic families. While a direct estimation of the overall entropy of language yielded values that varied for the different families considered, the relative entropy quantifying word ordering presented an almost constant value for all those families.

**Conclusions/Significance:**

Our results indicate that despite the differences in the structure and vocabulary of the languages analyzed, the impact of word ordering in the structure of language is a statistical linguistic universal.

## Introduction

The emergence of the human language faculty represented one of the major transitions in the evolution of life on Earth [1]. For the first time, it allowed the exchange of highly complex information between individuals [2]. Parallels between genetic and language evolution have been noticed since Charles Darwin [3] and, although there is still some debate, it is generally accepted that language has evolved and diversified obeying mechanisms similar to those of biological evolution [4]. There may even be evidence that all languages spoken in the world today originated from a common ancestor [5]. The extant languages amount to a total of some 7,000, and are currently divided into 19 linguistic families [6]. Within the Indo-European family some of the languages differentiated from each other not long after the end of the last glacial age [7], which pushes cross-family divergences far into prehistoric times. The evolutionary processes that acted since then have led to a degree of divergence that can make distantly related languages totally unintelligible to each other. Notwithstanding the broad differences between languages, it has been found that linguistic universals exist both at the level of grammar and vocabulary [8], [9], [10].

Written human languages encode information in the form of word sequences, which are assembled under grammatical and semantic constraints that create organized patterns. At the same time, these constraints leave room for the structural versatility that is necessary for elaborate communication [11]. Word sequences thus bear the delicate balance between order and disorder that distinguishes any carrier of complex information, from the genetic code to music [12], [13], [14]. The particular degree of order versus disorder may either be a feature of each individual language, related to its specific linguistic rules, or it may reflect a universal property of the way humans communicate with each other.

A rigorous measure of the degree of order in any symbolic sequence is given by the entropy [15]. The problem of assigning a value to the entropy of language has inspired research since the seminal work by Claude Shannon [16], [17], [18], [19]. However, to comprehend the meaning of the entropy of language it is important to bear in mind that linguistic structures are present at various levels of organization, from inside individual words to long word sequences. The entropy of a linguistic sequence contains contributions from all those different organizational levels.

In our analysis, we considered individual words as the most elementary units of linguistic information. Therefore, the first organizational level in a linguistic sequence is given by the distribution of frequencies with which different words are used. Zipf's law [20] states that if the word frequencies of any sufficiently long text are arranged in decreasing order, there is a power-law relationship between the frequency and the corresponding ranking order of each word. Moreover, this relationship is roughly the same for all human languages. Zipf's frequency-rank distribution, however, does not bear any information about the way in which words are ordered in the linguistic sequence, and would be exactly the same for any random permutation of all the words of the sequence. A second organizational level is then determined by the particular way in which individual words are arranged. Discriminating between the contributions of those two levels of organization can add relevant insights into statistical regularities across languages. The present paper is focused on assessing the specific impact of word ordering on the entropy of language. To that end, we estimated the entropy of languages belonging to different linguistic families. Our results show that the value of the total entropy depends on the particular language considered, being affected by the specific characteristics of grammar and vocabulary of each language. However, when a measure of the relative entropy is used, which quantifies the impact of word patterns in the statistical structure of languages, a robust universal value emerges across linguistic families.

## Results

### Empirical evidence for a quantitative linguistic universal

We analyzed eight corpora from five linguistic families and one language isolate, comprising a total of 7,077 texts. Texts were considered for the analysis as sequences of tokens. Each token was a word or, depending on the language, an equivalent unit of semantic content. In what follows, we will refer as ‘word’ to any of those basic linguistic units.

Due to the presence of long-range correlations in language [21], [22] it is not possible to compute accurate measures of the entropy by estimating block probabilities directly. More efficient nonparametric methods that work even in the presence of long-range correlations are based on the property that the entropy of a sequence is a lower bound to any lossless compressed version of it [15]. Thus, in principle, it is possible to estimate the entropy of a sequence by finding its length after being compressed by an optimal algorithm. In our analysis, we used an efficient entropy estimator derived from the Lempel-Ziv compression algorithm that converges to the entropy [19], [23], [24], and shows a robust performance when applied to correlated sequences [25] (see Materials and Methods).

For every text in the corpora two basic quantities were estimated. First, we computed the entropy of the original word sequence, *H*, which contains information about the overall order in the sequence. To quantify the contribution of word patterns that cannot be explained just by chance, we considered a random version of the text where linguistic order was absent. We achieved this by shuffling all the words in the original text in a totally random fashion. The typical entropy of the shuffled texts, denoted as *H_s_*, can be computed by direct methods (see Materials and Methods). By destroying linguistic structures at the level of word ordering, the degree of disorder in the sequence is increased. Thus, an estimation of the entropy of the disordered sequence typically yields a higher value. Therefore, the entropy of the original sequence can be written as 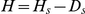, where the quantity *D_s_* is the decrease in entropy due to the ordering of words with respect to that contributed by their frequencies alone. The relative entropy *D_s_* can thus be used to quantify word ordering.

**Figure 1:**
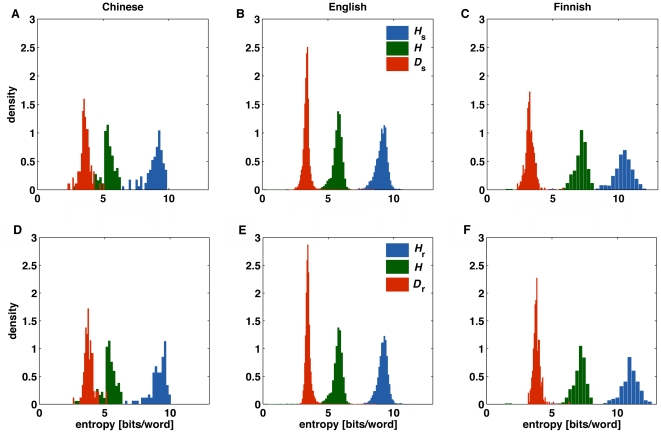
Entropy distributions for corpora belonging to three languages. Each panel shows the distribution of the entropy of the random texts lacking linguistic structure (blue); that of the original texts (green); and that of the relative entropy (red). The three languages: Chinese, English, and Finnish, were chosen because they had the largest corpora in three different linguistic families. In panels A, B, and C, the random texts were obtained by randomly shuffling the words in the original ones. In panels D, E, and F, the random texts were generated using the words frequencies in the original texts.

In Figure 1 we show the distribution of the entropy of individual texts obtained for three languages belonging to different linguistic families. In each of the upper panels, the rightmost distribution corresponds to the entropy of shuffled texts. The central distribution in each panel corresponds to the entropy of the original texts. This entropy contains contributions both from the words' frequencies regardless of their order and from the correlations emerging from word order. Note that the displacement between the two distributions is only a consequence of word ordering. Finally, the leftmost distribution in the upper panels of Figure 1 corresponds to the relative entropy *D_s_* between the original and shuffled texts in each language.

**Figure 2:**
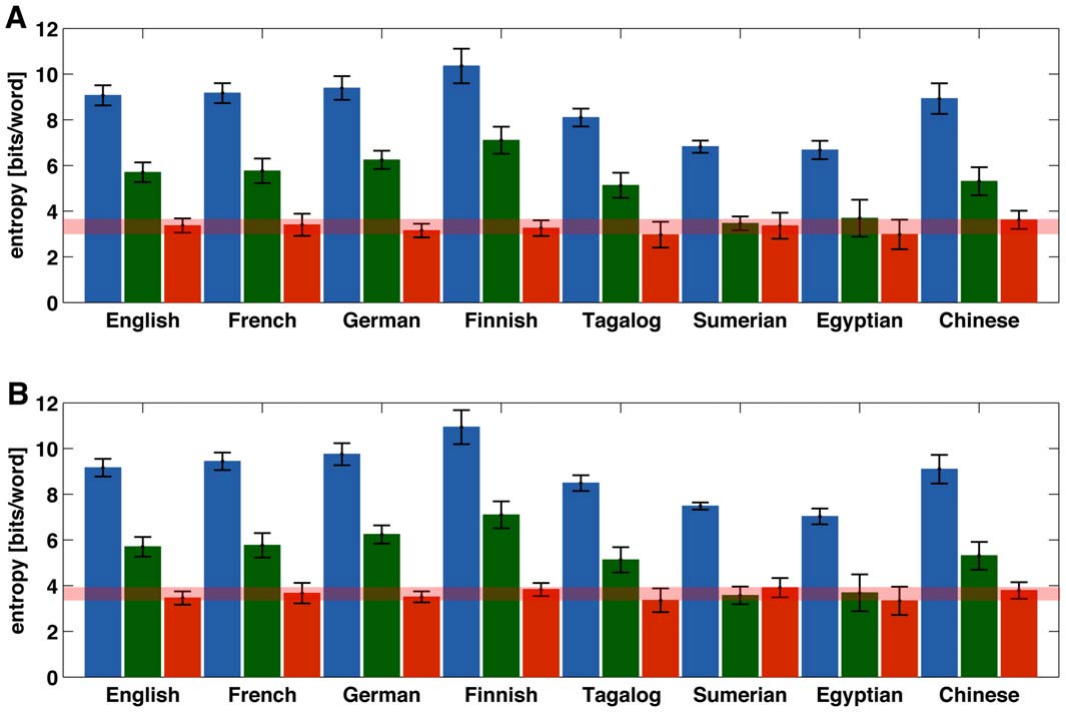
Entropy of eight languages belonging to five linguistic families and a language isolate (Indo-European: English, French, and German; Finno-Ugric: Finnish; Austronesian: Tagalog; Isolate: Sumerian; Afroasiatic: Old Egyptian; Sino-Tibetan: Chinese). For each language, blue bars represent the average entropy of the random texts, green bars show the average entropy of the original texts, and red bars show the difference between the entropies for the random and original texts. Error bars indicate the standard deviation within each corpus. The relative variability across all corpora, defined as the standard deviation divided by the mean of the entropy of the original texts was 0.23. (A) the random texts used to compute *H_s_* were obtained by shuffling the words' positions; the relative variability across all corpora was 0.14 for the random texts, and 0.07 for the corresponding relative entropy, *D_s_*. (B) the random texts were generated using the words' frequencies in the original texts. The relative variability across all corpora, was 0.15 for the random texts, and 0.06 for the corresponding relative entropy, *D_r_*.

For the three languages considered in Figure 1, the distributions of the relative entropy *D_s_* is narrower than those of the entropies *H* and *H_s_*, and they all seem to peak close to the same value. To verify whether this is the case for other languages as well, we computed the average of the three quantities, *H*, *H_s_*, and *D_s_*, for each of the eight corpora. The results are shown in Figure 2A. Due to grammar and vocabulary differences, the entropies of real and shuffled texts show large variability across corpora. However, their difference remains bounded within a narrow range around 3.3 bits/word across corpora and linguistic families (see also Table 1). For example, the language with the largest entropy for the random texts was Finnish, with average entropy of 10.4 bits/word while, at the other end, Old Egyptian had on average 7 bits/word. However, when we measured the relative entropy *D_s_* in both languages to quantify the impact of word ordering in their statistical structure we found 3.3 bits/word for Finnish and 3.0 bits/word for Old Egyptian. In other words, while the two languages showed a difference of almost 50% in the value of the entropy, they only differed by 10% in the value of the relative entropy. The relative variability across all corpora, defined as the standard deviation of entropies within each corpora divided by the mean entropy across corpora, was 0.14 for *H_s_*, 0.23 for *H*, and only 0.07 for the relative entropy *D_s_*. This suggests that beyond the apparent diversity found between languages, the impact of word ordering stands as a robust universal statistical feature across linguistic families.

**Table 1:** Estimated entropy values for each of the corpora.

Language	*H*_s_	*H*_r_	*H*	*D*_s_	*D*_r_
**English**	9.1	9.2	5.7	3.4	3.5
**French**	9.2	9.4	5.8	3.4	3.6
**German**	9.4	9.8	6.2	3.1	3.5
**Finnish**	10.4	10.9	7.1	3.3	3.8
**Tagalog**	8.1	8.5	5.1	3.0	3.4
**Sumerian**	6.8	7.5	3.5	3.4	3.9
**Old Egyptian**	6.7	7.0	3.7	3.0	3.3
**Chinese**	8.9	9.1	5.3	3.6	3.8

For each language the table shows the corresponding entropy values in bits/word. The data correspond to the texts that stood the convergence test described in Materials and Methods.

### Universality of the Kullback-Leibler divergence

The analysis in the previous section shows that a measure of relative entropy between a real text and a disordered version of it where word order has been destroyed presents an almost constant value across different linguistics families. We also considered another mechanism to neglect linguistic structure in the texts that makes it possible to relate the relative entropy to the Kullback-Leibler divergence between real and random texts, and thus set the analysis within the framework of standard information theory. As before, the random text was a sequence of the same length as the original one. Now, however, each place in this sequence was assigned a word chosen at random with a probability given by that word's frequency in the original text. On the average over many realizations of the sequence, the frequencies of each word in the original text and in its random version were the same but, in the latter, word ordering was determined by chance and lacked any linguistic origin. All the possible random sequences generated from the same original text defined an ensemble to which an entropy measure can be assigned. That entropy, which we denote as *H_r_*, can be computed directly from the Zipf's distribution of the original text (see Materials and Methods). The values of *H_r_* obtained for the texts in our corpora were similar to the values obtained for entropy of the disordered texts, *H_s_*, as can be seen in the lower panels of Figure 1, and comparing with the upper panels of the same figure. Moreover, it can be shown that for the limit of very long texts both *H_r_* and *H_s_* become identical (see Materials and Methods).

Besides allowing a direct connection with the formalism of information theory, this second model of the random texts rigorously neglects all correlations between word positions. Within this model, in fact, the probability of occurrence of sequence of words is given as the product of the normalized frequencies of the individual words (see Materials and Methods). We can also relate the values of the entropy of the random texts in lower panels of Figure 1 to the lexical diversity of the different languages. For instance, highly inflected languages, like Finnish, have very diversified vocabularies due to the multiplicity of word forms that derive from a common root. This leads to a relatively flat Zipf's distribution with higher average entropy [26]. On the other hand, less inflected languages such as English tend to have a steeper Zipf's distribution with lower entropy.

Proceeding in a similar way as in the previous section we computed the difference 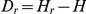, which is an estimation of the relative entropy, or Kullback-Leibler (KL) divergence, between the original and random texts [15] (see Materials and Methods). Repeating the analysis using this measure, we found that the KL divergence across all the linguistic families considered remains almost constant around 3.6 bits/word, as shown in Figure 2B. This suggests that our main finding of a linguistic universal related to the quantification of word ordering does not depend on the precise way in which linguistic order is neglected or destroyed.

### Simplified language models

**Figure 3:**
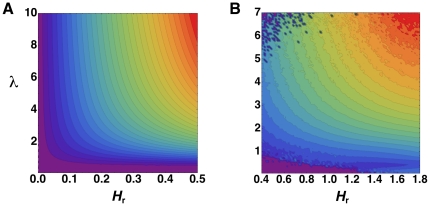
Impact of word correlations in simplified models of language. Panels show curves of constant Kullback-Leibler divergence, *D_r_*, as a function of both the entropy of the random sequence, *H_r_*, and the correlation length between words, *λ*. Colors towards the violet represent lower values of the divergence *D_r_*. The divergence quantifies the impact of word correlations in the overall entropy of the texts. (A) Divergence *D_r_* for a two-word Markovian model of language computed analytically as described in Materials and Methods. (B) Average divergence corresponding to a numerical simulation of 10^11^ realizations of a four-word Markovian language model.

In order to gain insight on the origin and meaning of the common value of the relative entropy, *D_r_*, across linguistic families, we studied a few simplified models where the interplay between vocabulary and correlation structures can be understood either analytically or numerically. We first studied a minimalist model that can be completely solved analytically. It describes a language with only two words as a first order Markov process. In this simple case, the Zipf's distribution is completely determined by the overall probability of occurrence of one of the two words, which we call *ρ*. The other parameter is the correlation length between words in a linguistic sequence, *λ*. Once the parameter *ρ* is fixed, the entropy *H_r_* can be computed. Details of the model are given in the Materials and Methods section. In Figure 3A we show a contour plot of the KL divergence as a function of the entropy of the random sequence, *H_r_*, and the correlation length. The contour lines correspond to the curves of constant *D_r_*. This shows that in the two-word language model the constraint of maintaining a constant value of the KL divergence requires that an increase in correlation length is balanced by a decrease in the entropy of the random sequence *H_r_*.

The same behavior was found in a *K*-word language Markov model, defined by 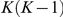 independent parameters (see Materials and Methods for details). Despite the fact that for 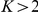 the model is not completely determined by the two parameters *λ* and *ρ*, it is still possible to evaluate the correlation length and the entropy of the Zipf's distribution. In Figure 3B, we present a contour plot of the KL divergence as a function of those two quantities for 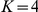. Each value in the plot represents an average of the KL divergence over many realizations of a language for the corresponding values of *λ* and *ρ*. Overall, the plot shows the same pattern found for the two-word language model in Figure 3A. Similar results, not presented here, were obtained for 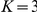. In *K*-word languages with 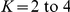 therefore, keeping the KL divergence constant requires that the entropy of the random sequence increases when the correlation length decreases, and vice versa.

Numerical analysis of *K*-word language Markov models becomes prohibitively difficult for 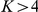. However, we can still use the insight gained from those models to test whether similar behavior occurs in real languages. For the latter, the computation of *Hr* is performed as discussed in the preceding section. The estimation of the correlation length for words in real language is, on the other hand, a difficult task, due to the limited sampling of joint occurrences. Moreover, correlations in language decay as power-law functions [21], [27], which means that they have significant values over considerable lengths, spanning up to hundreds or thousands of words. In order to provide a quantitative measure of correlations in real language, we used the Detrended Fluctuation Analysis technique for estimating the fluctuation exponent *α* [28], [29], [30]. This exponent is closely linked to the structure of correlations (see Material and Methods for details): the larger *α* the slower the decay of correlations.

**Figure 4:**
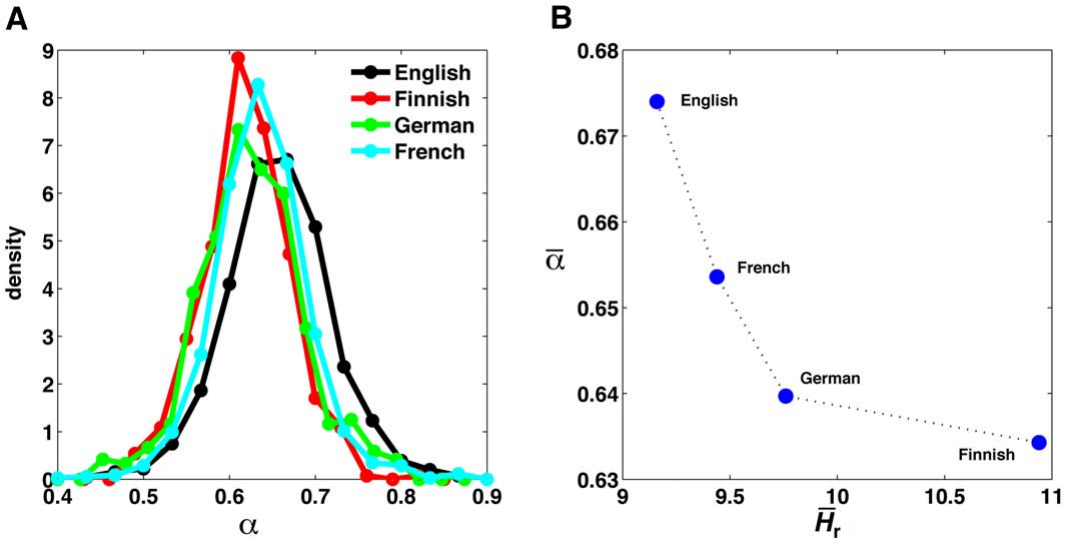
Word correlations and entropy in real languages. (A) Normalized histograms of the fluctuation exponent *α* computed using Detrended Fluctuation Analysis (see Materials and Methods) for four languages. The medians of the distributions are statistically different (*p*<10^−5^, Mann-Whitney *U*-test computed over all possible pairs). (B) Average fluctuation exponent, 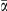, as a function of the average entropy of the random texts, 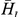, for the same languages as shown in panel A.

We calculated the fluctuation exponent *α* for all the texts in the corpora. Its distribution was only slightly variable across languages, showing large overlapping areas. Thus, as a test for the statistical significance of their differences, we estimated significance values *p* for the medians of each pair of distributions, and only kept those for which the null hypothesis of equal medians could be rejected (*p*<10^−5^, Mann-Whitney *U*-test [31]). In Figure 4A we present the distributions for the four languages that passed the statistical test. Figure 4B shows the fluctuation exponent *α* as a function of average entropy of the random texts 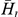 for each of the languages considered in Figure 4A.

Bearing in mind the relation between the exponent *α* and the decay of correlations, the plot in Figure 4B can be compared with the contour plots of Figure 3. Real languages, for which the KL divergence is approximately constant (see Figure 2B), define a contour line with the same interdependency between correlation and entropy as observed in the simplified model languages.

## Discussion

We estimated the entropy for a large collection of texts belonging to eight languages from five linguistic families and one language isolate. Linguistic differences are reflected in variations of the value of the entropy across languages. In principle, for some of the languages considered, variability of the direct entropy measures could be related to the specific stylistic make up of each dataset. However, a large variability was also observed within the group of European languages, which were homogeneous in terms of styles, consisting mostly of literature and some technical texts.

In order to assess the impact of correlations deriving from word ordering, we studied the differences between the entropy obtained from the original linguistic sequences and the entropy of random texts lacking any linguistic ordering. While the entropy of a symbolic sequence is well defined in the limit of infinite length, we only considered texts for which our entropy estimators showed convergence. This measure of relative entropy yielded an almost constant value for all languages considered. This was observed both when linguistic order was destroyed by disordering the words and when a more formal model was used in which correlations between words are ignored. Therefore, our evidence suggests that quantitative effect of word order correlations on the entropy of language emerges as a universal statistical feature.

To understand the meaning of this finding we addressed two simplified models of language in which we had control on their structure. We estimated the impact of correlations in the structure of these model languages as a function of the diversity of basic symbols, represented by *H_r_*, and a measure of the strength of correlations among the symbols. At variance with real languages, these simplified models based on Markov processes show a correlation between words that decays exponentially rather than as a power law. However, they provide an ideal heuristic framework to isolate the interplay between symbol diversity and correlation length in symbolic sequences. The results showed that in order to keep constant the relative entropy, as is the case in real languages, an inverse relationship must exist between the correlation length and the entropy of the random text *H_r_*. Remarkably, real languages showed the same overall dependency, with languages with higher entropy *H_r_* having correlations with a faster decay, and vice versa.

Quantifiable statistical features shared by languages of different families are rare. The two best known quantitative linguistic universals are Zipf's law [20] and Heap's law [32], which refer to the statistics of word frequencies. The property disclosed in this paper, on the other hand, is the first that addresses the finer level of word ordering patterns.

During their evolution, languages underwent structural variations that created divergences in a way not very different from biological evolution [4]. This may explain the variations in parameters like the correlation length and the symbol diversity found for different languages. However, our analysis shows that the evolutionary drift was constrained to occur keeping the relative entropy almost constant. Across all the families considered, the variability of the entropy was almost 400% larger than the variability observed in the relative entropy. Thus, according to our results, the relative entropy associated with word ordering captures a fundamental quantitative property of language, which is common to all the examples analyzed in this paper. More generally, these results suggest that there are universal mechanisms in the way humans assemble long word sequences to convey meaning, which may ultimately derive from cognitive constrains inherent to the human species.

## Materials and Methods

### 1. Estimation of the relative entropy in symbol sequences

Let us represent any generic word in the text sequence by *x_i_*. Then, any text segment of *n* words in length can be represented as 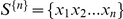. We assume that each word belongs to a given lexicon *W*, 
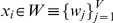
.

To compute the entropy of the shuffled text, let us note that the number of ways in which the words can be randomly arranged is given by
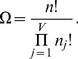
(1)Since any of the possible permutations of the word's positions has the same probability of occurrence, the entropy per word of the shuffled texts will be given by
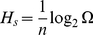
(2)


### 2. Quantification of the impact of word correlations using the Kullback-Leibler divergence

Let 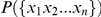 be the probability of occurrence of a given word sequence of length *n*. In particular, 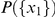 is simply the normalized frequency of occurrence for a single word. Thus, if we now consider a random version of the text in which there are no correlations in the ordering of words, the probability of any given sequence of length *n* is given by the product of the single-token marginal probabilities of the original text, 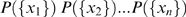. The entropy per word of the original text is then given by the following expression:
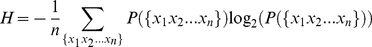
(3)In a similar way, the entropy of the random text is given by:
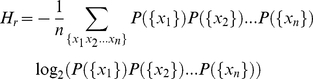
(4)In both equations we assumed that *n* is sufficiently large as to account for all possible correlations in the sequences of the original text. For sequences with unbounded correlations the limit of *n* going to infinity must be taken.

The difference of the entropies defined above, 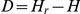, is a measure of the relative entropy or Kullback-Leibler divergence between the probability distributions that describe the random and the original texts (Cover and Thomas, 2006). By subtracting Equations 4 and 3, we find that the Kullback-Leibler divergence reads,
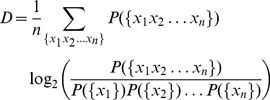
(5)It is straightforward to verify that the right-hand side of Eq. 5 is indeed the difference 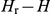 as defined above. The crucial step is noting that, since
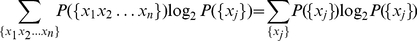
(6)for all *j*, the entropy of the random text can also be written as
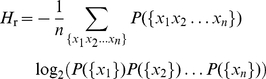
(7)The entropy of the original texts accounts for contributions from the ordering of words and the frequency of occurrence of those words. Instead, in the random texts only the latter contribution is present. Thus, their difference bears information about patterns in word ordering that are beyond chance and are due to correlations in written language.

It is not difficult to show that for long texts, Eq. 2 and Eq. 4 yield very similar values, and become identical in the limit of infinitely long texts. To show this, one just needs to expand the logarithm of the factorials using Stirling's approximation [33] and rearrange terms.

### 3. Entropy estimation based on compression algorithms

Direct methods of entropy estimation based on the computation of block probabilities have proved extremely difficult to apply to linguistic sequences due to the exponential explosion in the number of parameters to estimate from finite data. This is particularly true in the case of human language, given their long-range correlations [21], [22], [34]. An alternative approach is provided by non-parametric methods that do not rely on any *a priori* assumption about the correlation structure of the sequences. In particular, we used methods based on the Lempel-Ziv compression algorithm that converge to the entropy even in the presence of long-range correlations [19], [23], [35].

An important property of the entropy is that it is a lower bound to the length of any lossless compressed version of a symbolic sequence [15]. Thus, in principle, it is possible to estimate the entropy of a symbolic sequence by finding the minimum length to which it can be compressed without information loss. However, instead of using an algorithm to compress the linguistic sequences, we used an improved estimator based on the principles of the Lempel-Ziv compression algorithm that shows a faster conversion to the entropy. The details of the particular implementation, and its application to estimate the entropy of English, are described in [19]. Here, we briefly review the basic procedure.

Let us consider a whole symbolic sequence of length *n* as 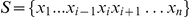, where *i* denotes any position inside the sequence. For every position *i*, there is a length, 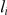, corresponding to the shortest contiguous subsequence that starts at position *i*, and does not appear in any continuous subsequence starting anywhere between position 1 and 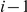. For instance, consider the following alphabetical sequence: CDABCDEABCZ; at position 8, the shortest mismatch is 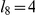. After parsing the whole sequence, the resulting series 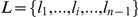 will contain information about the redundancy in it. This procedure is at the heart of the Lempel-Ziv compression algorithm [23] and the entropy estimation method used in our analysis. In particular, it can be shown that under certain general conditions the entropy *H* of the symbolic sequence can be estimated as follows [19],
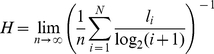
(8)Although the limit cannot be attained in practice, we checked convergence by computing the entropies from two halves of the text and then comparing to the entropy of the whole text. Only texts for which there was a maximum discrepancy of 10% in the relative entropy estimation of the whole text and its halves were accepted for the analysis. We tested that there was no difference in the conclusions by either taking the threshold at 5% or 20%, thus indicating that results are robust.

### 4. Simplified models of language

Markov processes have been used extensively in language modelling. They have the advantage of allowing a systematic control on the complexity of the correlation structure, and have often been used as an approximation to complex natural processes.

### A two-word Markovian language

This minimal model describes language as a first order Markov process with a vocabulary of two words.

The model can be characterised by only two parameters. Let the vocabulary of the language be 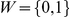. The transition matrix (grammar) of the Markov process is given as follows:
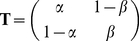
(9)where 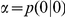 and 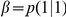. To simplify the notation denote by 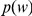 the probability of finding the symbol 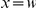 anywhere in the sequence. In the same way, 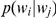 is the conditional probability of finding symbol 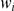 at a particular position in the sequence given that it was preceded by symbol 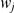.

As a more convenient pair of parameters to describe the model language we choose the correlation length of the sequence and the rate of occurrence of the word ‘1’. This overall rate determines the shape of the Zipf's distribution for the language and thus is related to the diversity of the vocabulary. It can be computed as the unconditional probability of the word ‘1’ in the sequence, 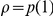. Since 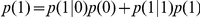, we have
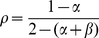
(10)The correlation length can be related to the transition probabilities by computing the autocorrelation function for the process,
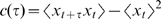
(11)Since the only variable pair contributing to the correlation is the 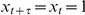, we just need to compute the 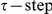 probability 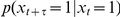. Thus, the correlation function becomes
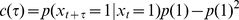
(12)We find
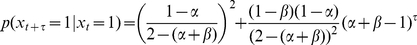
(13)Then
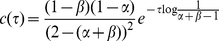
(14)from which the correlation length is
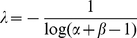
(15)Finally, using the equations for *λ* and *ρ*, we can write,
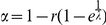
and
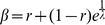
(16)In this way we related the transition probabilities to the correlation length *λ*, and the symbol diversity *ρ*.

### Kullback-Leibler divergence for the two-word model

The entropy rate of any ergodic process can be computed as the following limit:
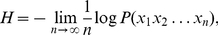
(17)where we used *H* to designate the entropy of the original sequence. If the process is first order Markov, we have
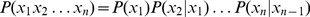
(18)Thus,
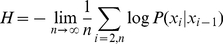
(19)where we dropped the term for *i* = 1 since it does not contribute to the limit. We can group the terms in the sum above and write the entropy for the Markov process in terms of the transition probabilities and the symbol rates.
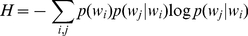
(20)where 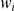 are the vocabulary symbols, or words. In the two-word language model the transition probabilities are given in the matrix 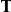 in Eq. 9. Equation 20 can then be written more clearly if we introduce the column-wise entropies of the Markov transition matrix:
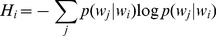
(21)The entropy of the original sequence can be written as
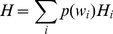
(22)For the specific case where the transition probabilities are given by the matrix **T**, we have for the column-wise entropies
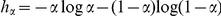


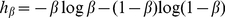
(23)where *α* and *β* are given in terms of the correlation length and symbol diversity through Eq. 16.

Then, the entropy of the two-word Markov sequence takes the following form:
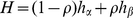
(24)Finally, the entropy of the random sequence can be easily computed from the *rate* parameter *ρ*, as
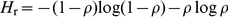
(25)Therefore, the Kullback-Leibler divergence is computed as 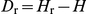.

### *K*-word Markovian language model

A 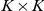 transition matrix contains 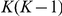 parameters since there are 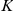 normalization conditions for its columns. In general, the stationary distribution of the Markov process is the normalized eigenvector of the matrix 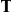 corresponding the largest eigenvalue of the transition matrix, which is always unity for a Markov process. From the stationary distribution the entropy of the random sequence, 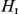 follows immediately, and Eq. 21 and Eq. 22 can be used to obtain *H* for the model language.

An estimation of the correlation length can be obtained by considering the properties of the 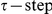 transition matrix 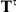 in the case of the *K*-word language model. From the spectral decomposition of the matrix 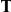, we have
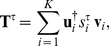
(26)where 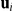, 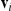, and 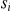 correspond respectively to the left and right eigenvectors and eigenvalues of the matrix 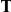. By rearranging the sum in Eq. 26, we have
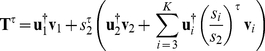
(27)Since all the ratios 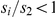 for 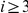, for large 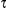 the decay of the second term of the right hand side in Eq. 27 is determined by the second eigenvalue term 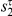. This holds for all the elements of the matrix 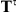. Then, all the correlation functions also decay as 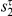 or, equivalently, as 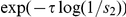. Therefore, we can define a correlation length for the *K*-word language models as follows:
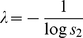
(26)The dimension of the parameter space to explore grows as 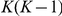, thus making it very difficult to analyze languages with large values of *K*. For instance, acceptable statistics for 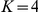 required the realization of 10^11^ transition matrices.

### 5. Detrended Fluctuation Analysis

Correlations in language are known to be of the power-law type [21], [27], decaying as 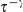. Then, the smaller *γ* the slower the decay of the correlation. It is possible to estimate *γ* using the method of Detrended Fluctuation Analysis [28], [29]. In particular, the fluctuation exponent *α* is related to the correlation exponent *γ* by a simple linear relationship, 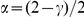. Thus, the slower the decay of the correlation strength (smaller *γ*) the larger *α*.

Here we used the word as the minimum unit of information. The mapping of texts onto time series is achieved by replacing every word by its rank in the in a list of the words used in the text ordered by decreasing frequency. Thus, the most frequent word is replaced by ‘1’, the second most frequent by ‘2’, and so on [34].

### 6. Description of the corpora

All but the Sumerian and Egyptian texts were obtained from *Project Gutenberg* (www.gutenberg.org). The Indo-European and Finnish texts comprised a mixture of literary, scientific, historical, and philosophical books. The Chinese texts were a collection of literary and philosophical books from different periods from antiquity to the present. The Tagalog corpus contained a variety of literary texts including poetry. The Old Egyptian texts were obtained from the page maintained by Dr. Mark-Jan Nederhof at the University of St Andrews (www.cs.st-andrews.ac.uk/~mjn/egyptian/texts/) as transliterations from the original hieroglyphs. The Sumerian texts were downloaded from *The Electronic Text Corpus of Sumerian Literature* (www-etcsl.orient.ox.ac.uk/) and consisted of transliterations of the logo-syllabic symbols. In the case of Chinese, Old Egyptian, and Sumerian, the basic linguistic units that we referred to as *words* were respectively, logograms, hieroglyphs, and logo-syllables. Details of text sizes for each corpus can be found in Table 2.

**Table 2:** Details of the analized corpora.

Language	Number of texts	Mean text length	Median text length	Shortest text	Longest text
**English**	5112	67206	48904	1347	1267490
**French**	417	68727	59338	872	330339
**German**	999	44280	27820	2542	950371
**Finnish**	392	30991	23355	2095	159444
**Tagalog**	47	20086	11506	2953	209789
**Sumerian**	5	4766	4766	4246	5286
**Old Egyptian**	4	3284	3853	1101	4328
**Chinese**	101	109300	41953	1106	771917

For each language analyzed the table shows the size of the corpora in number of texts and the data specifying the average, median and absolute ranges of text sizes measured in number of words. As in Table 1, the data correspond to the final set of texts used in the analysis.
